# *Neisseria* genomics: current status and future perspectives

**DOI:** 10.1093/femspd/ftx060

**Published:** 2017-06-07

**Authors:** Odile B Harrison, Christoph Schoen, Adam C Retchless, Xin Wang, Keith A Jolley, James E Bray, Martin C J Maiden

**Affiliations:** 1 Department of Zoology, University of Oxford, Oxford OX1 3SY, UK; 2 Institute for Hygiene and Microbiology, University of Würzburg, Würzburg 97080, Germany; 3 Centers for Disease Control and Prevention, Atlanta, GA 30333, USA

**Keywords:** next-generation sequencing, *Neisseria meningitidis*, *Neisseria gonorrhoeae;* Omics analyses

## Abstract

High-throughput whole genome sequencing has unlocked a multitude of possibilities enabling members of the *Neisseria* genus to be examined with unprecedented detail, including the human pathogens *Neisseria meningitidis* and *Neisseria gonorrhoeae*. To maximise the potential benefit of this for public health, it is becoming increasingly important to ensure that this plethora of data are adequately stored, disseminated and made readily accessible. Investigations facilitating cross-species comparisons as well as the analysis of global datasets will allow differences among and within species and across geographic locations and different times to be identified, improving our understanding of the distinct phenotypes observed. Recent advances in high-throughput platforms that measure the transcriptome, proteome and/or epigenome are also becoming increasingly employed to explore the complexities of *Neisseria* biology. An integrated approach to the analysis of these is essential to fully understand the impact these may have in the *Neisseria* genus. This article reviews the current status of some of the tools available for next generation sequence analysis at the dawn of the ‘post-genomic’ era.

## Teaser

This manuscript discusses how large volumes of genome data available can be organised, analysed and used to best advantage and has a particular focus on the bacterial pathogens: *Neisseria meningitidis* and *Neisseria gonorrhoeae*.

## INTRODUCTION

In the late 1970s, methods were developed to sequence DNA by chain termination or fragmentation techniques. These revolutionised biology by enabling nucleotide sequences from complete genes to be determined and provided the tools for biologists to examine genetic variation (Sanger, Nicklen and Coulson 1977; Maxam and Gilbert 1980). Around 20 years later, the Sanger sequencing method was used to establish high-quality complete reference genomes for a number of organisms including humans and many important bacterial pathogens. The first pathogens to be sequenced were *Haemophilus influenzae* and *Mycoplasma genitalium* (Fleischmann *et al.*^1995^; Fraser *et al.*^1995^) providing for the first time an insight into the complete genomes of living organisms (Forde and O’Toole 2013). Following this, further improvements in sequencing techniques were made with the use of approaches such as paired-end sequencing and the whole genome shot-gun approach (Roach *et al.*^1995^; Weber and Myers 1997). These techniques paved the way for the sequencing of *Neisseria meningitidis* genomes in 2000, from the isolates Z2491 (serogroup A, accession number: NC_0 03116) and MC58 (serogroup B, NC_0 03112) (Parkhill *et al.*^2000^; Tettelin *et al.*^2000^). This was followed in 2003 with the genome from *N. gonorrhoeae* FA1090 (http://www.genome.ou.edu/gono.html, NC_0 02946) and *N. lactamica*, 20–06, in 2010 (FN995097) (Bennett *et al.*^2010^).

In this first phase of genome sequencing, methodologies based on the Sanger sequencing technology dominated; however, these techniques were laborious and time consuming. As a result, sequencing focussed on model organisms with only a few representative isolates from, for example, species of medical importance sequenced. In contrast to conventional sequencing methods, next-generation sequencing (NGS) technologies have the capacity to produce thousands to many millions of sequencing reactions in parallel, without the need for electrophoresis or bacterial cloning and provide a low-cost, high-throughput alternative to conventional Sanger sequencing approaches. The first NGS technology to be released, in 2005, was the pyrosequencing method by 454 Life Sciences (now Roche) with those from the Solexa/Illumina sequencing platform commercialised 1 year later in 2006. In 2007, Sequencing by Oligo Ligation Detection (SOLiD) was released by Applied Biosystems (now Life Technologies) with the Personal Genome Machine (PGM) released in 2010 by Ion Torrent (also now Life Technologies). Initially, these technologies produced short nucleotide (nts) read lengths averaging 35 to 110 nts (Illumina and 454, respectively); however, these have been extended with read lengths of up to 300 nts now possible using the Illumina MiSeq platform. Finally, the Pacific Biosciences (PacBio) sequencing platform PacBio RS II, released in 2011, is able to produce long reads of up to 20 000 nts enabling complete finished bacterial genomes to be obtained relatively rapidly and inexpensively (van Dijk *et al.*^2014^).

At the time of writing, the introduction of NGS technology provided the capacity to sequence thousands of bacterial isolates. Current challenges therefore now lie in the handling of the large amount of data generated by these technologies and how these should be organised, analysed and applied to best advantage. This review will explore these issues using the genus *Neisseria* as an exemplar. The organisation, analysis, application and impact of NGS data in *Neisseria* research will be discussed with a particular focus on the human pathogens *N. meningitidis* and *N. gonorrhoeae.* This review is based on discussions at the *Neisseria* genomics workshop which took place at the International Pathogenic *Neisseria* Conference in 2016 in Manchester, UK.

## ORGANISATION AND ANALYSIS OF *NEISSERIA* NGS DATA

**Figure 1.**
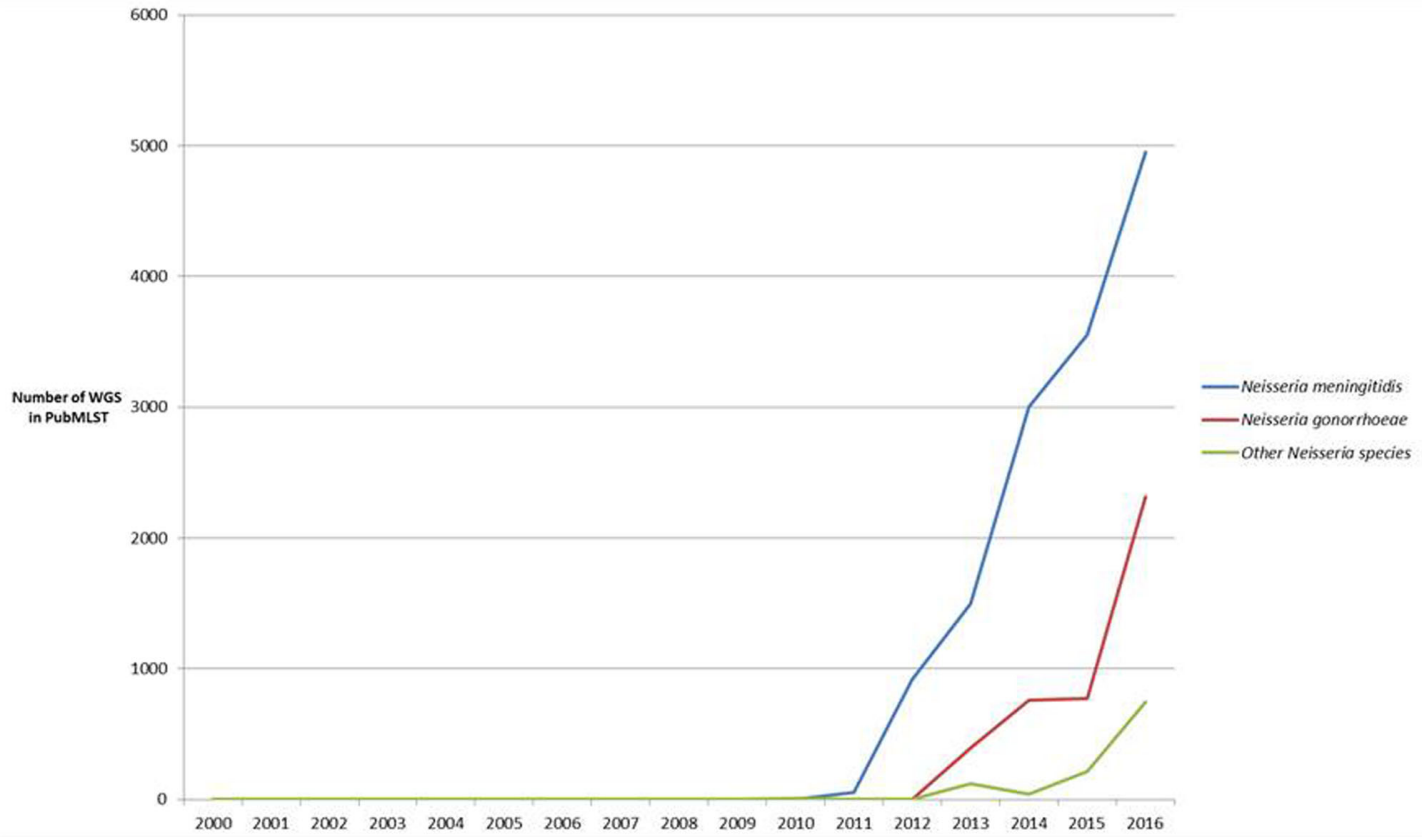
Cumulative number of assembled WGS data deposited in PubMLST.org/neisseria. Graph depicting cumulative whole genome sequence data available in PubMLST dating from 2000 to the end of 2016. Blue lines depict *N. meningitidis* WGS data starting with relatively few isolates in 2010 and peaking at 5000 isolates at the end of 2016; red depicts *N. gonorrhoeae* WGS data peaking at over 2000; green all of the other *Neisseria* species including *N. lactamica, N. subflava, N. polysaccharea, N. cinerea, N. mucosa, N. dentiae, N. musculi, N. oralis, N. bacilliformis* and *N. elongata.*

The genus *Neisseria* includes the human pathogens *N. meningitidis* and *N. gonorrhoeae*, which are significant causes of morbidity and mortality worldwide. As a result, isolates of these species have been extensively sequenced since 2000, with NGS generating an abundance of whole genome sequence (WGS) data. At the time of writing, WGS data from *N. meningitidis* outnumbered any of the other *Neisseria* species, with WGS from 13 985 meningococci available (Fig. 1), although the availability of WGS from *N. gonorrhoeae* was rapidly increasing at the time of writing, a consequence of the rise in antimicrobial-resistant gonococci which has provided a renewed incentive to resolve this global health problem (Grad *et al.*^2014^; Ezewudo *et al.*^2015^; De Silva *et al.*^2016^; Didelot *et al.*^2016^).

**Figure 2.**
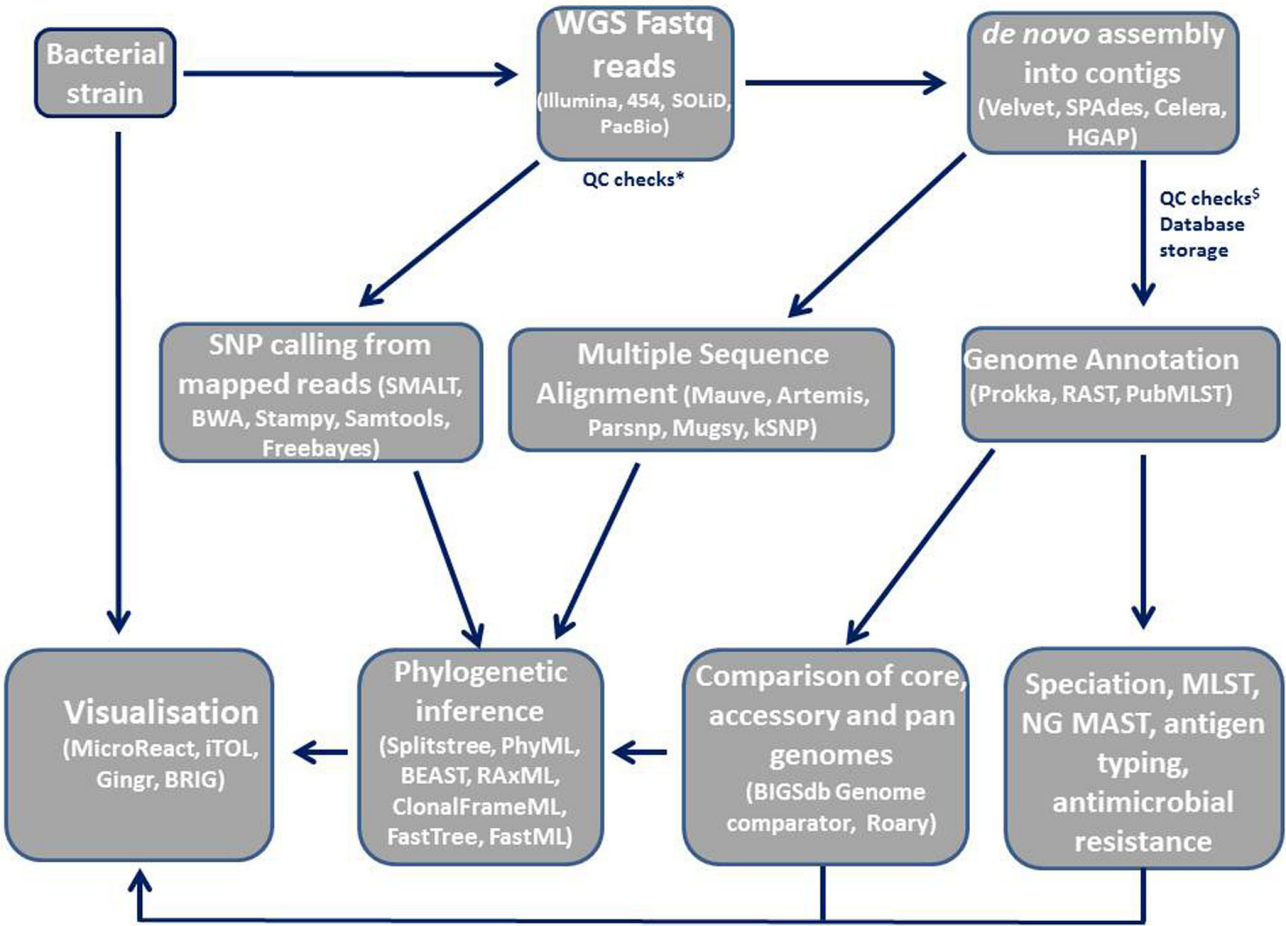
NGS and analysis pipelines. Most common tools used for the analysis of WGS data. QC checks*: quality control before *de novo* assembly: poorly identified bases, low quality sequences and/or contaminants such as adaptors. QC checks^$^: quality control checks after assembly for mixed samples and bacterial contamination.

The volume of data generated from WGS can rapidly overwhelm computer storage space with, for example, an average of 150 Mb of data produced per bacterial genome. Such data are therefore rarely stored locally, and are for the most part deposited in the European Nucleotide Archive (ENA) or the National Center for Biotechnology Information (NCBI). A multitude of tools and methodologies are subsequently available for WGS analysis, many of which are not mutually exclusive and are largely dependent on the research question investigated (Fig. 2).

### The Bacterial Isolate Genome Sequence Database (BIGSdb) platform

Once assembled *de novo* into contiguous stretches of nucleotide sequences (contigs) using assembly software such as Velvet or SPAdes (Zerbino 2010; Bankevich *et al.*^2012^), WGS data become more manageable and easy to store with the added benefit that these data can be repeatedly referred to and re-analysed thus generating reproducible and readily accessible data. *Neisseria* research has benefited from the availability of the PubMLST website (https://pubmlst.org/neisseria) which was initially set up to catalogue the seven multi-locus sequence type (MLST) allelic variants together with isolate provenance metadata (Jolley and Maiden 2013). This has been a continually expanding and well-accessed resource including, at the time of writing, nearly 42 000 isolate records with over 19 000 of these comprising genomes assembled from WGS data and 12 550 sequence types (ST). In addition, isolate records are hyperlinked to raw sequencing data at the ENA.

**Figure 3.**
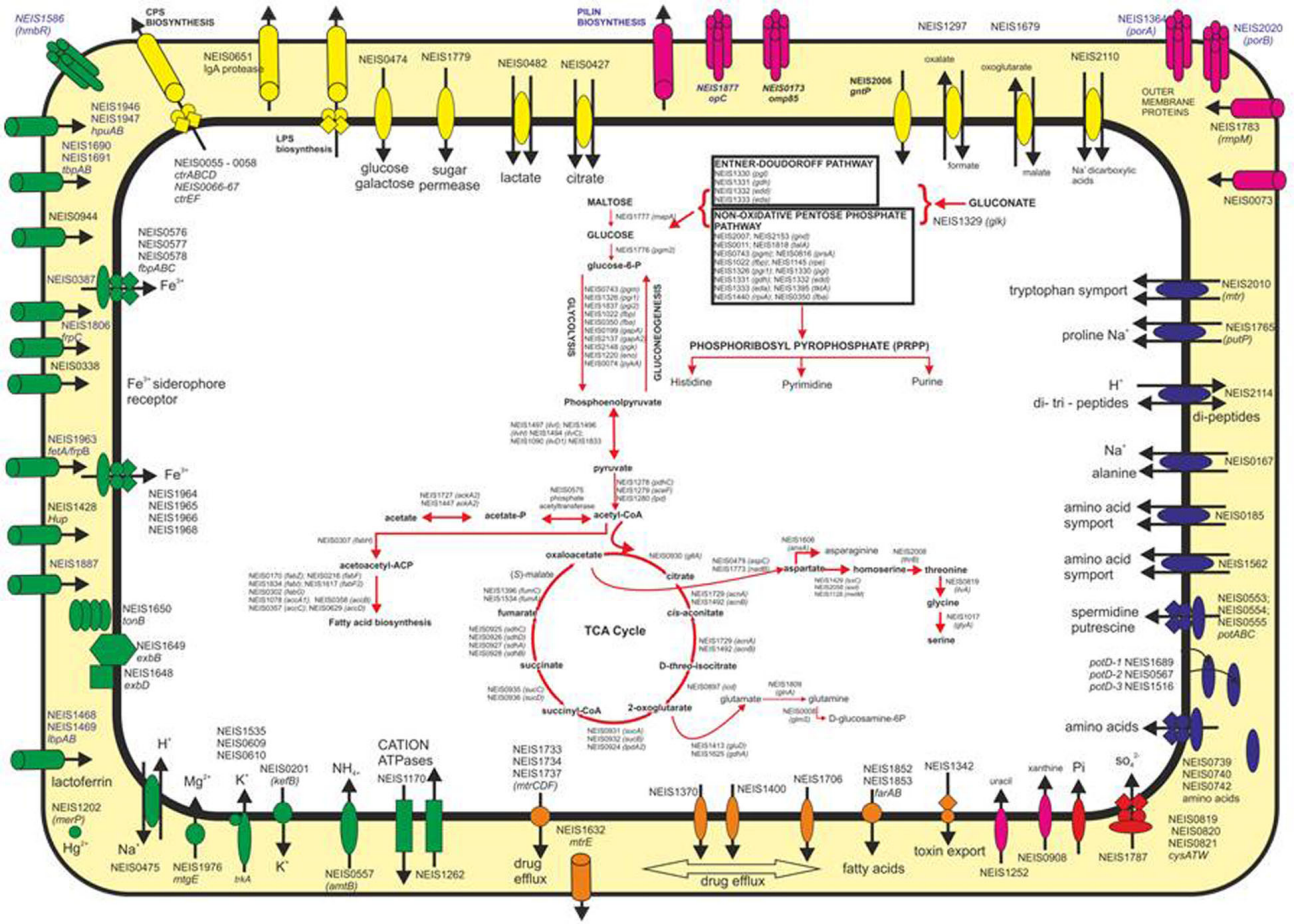
NEIS loci defined in PubMLST. Representative figure of a bacterial cell and some of the loci found across *Neisseria* species. Green structures represent acquisition receptors; pink indicate antigens; orange import/export elements; blue amino acid import/export; yellow sugar import/export; various metabolic pathways are also indicated. Additional loci have been defined in PubMLST. Figure based on the study by Tettelin *et al.* (2000).

In 2012, PubMLST implemented the Bacterial Isolate Genome Sequence Database (BIGSdb) platform, which was developed to facilitate the flexible storage and exploitation of NGS data (Jolley and Maiden 2010). The platform consists of two kinds of database: (i) a sequence definition database that contains sequences of known alleles for loci as well as allelic profiles for specific schemes such as MLST and (ii) an isolate database that contains isolate provenance and other metadata along with nucleotide sequences associated with that isolate. Sequence definitions have now been established for over 2600 protein-encoding genes, annotated as loci with the NEIS prefix and the majority of these have been organised into schemes dependent on function. Many of these loci are shared across the *Neisseria* genus permitting WGS data from all *Neisseria* species to be annotated, including both meningococci and gonococci (Fig. 3).

Using gene discovery tools including Prokka and/or RAST (Aziz *et al.*^2008^; Seemann 2014), the catalogue of defined loci in PubMLST is continually increased allowing, for example, the entire set of genes found in all isolates from any species to be determined (known as the pan genome). As a result, loci found in mobile genetic elements such as plasmids, the meningococcal disease-associated island as well as secreted toxins associated with *maf* genomic islands can be annotated in WGS data (Jamet *et al.*^2015^; Meyer *et al.*^2016^). In addition, locus definitions are not limited to coding sequences, such that any stretch of nucleotide or translated peptide sequence can be defined including intergenic regions, antigenic epitopes, small regulatory RNAs and promoters. To distinguish such features from ORFs, promoter loci in the PubMLST *Neisseria* database have been assigned the ‘pro’ prefix (for promoter) followed by the corresponding locus suffix for the adjacent gene to differentiate them from coding sequences e.g. *^pro^*NEIS1635 (*mtrR*). Point mutations located upstream of the *mtrCDE* operon and/or the *mtrR* promoter region upstream of the MtrR transcriptional regulator are associated with increased expression of the MtrCDE efflux pump leading to antimicrobial resistance in gonococci (Hagman and Shafer 1995; Lucas *et al.*^1997^; Ohneck *et al.*^2011^). Annotation and comparison of this promoter region in combination with loci implicated in antimicrobial resistance therefore allows antimicrobial-resistant genotypes to be determined (Harrison *et al.*^2016^). Small non-coding regulatory RNAs (ncRNAs) have also been defined in PubMLST including *nrrF* (Mellin *et al.*^2010^) with plans to include additional ncRNAs in the future as more of these are identified.

WGS data deposited in PubMLST are automatically annotated for any of the defined loci, updating isolate records with allele numbers. New alleles are automatically identified if they exhibit ≥98% sequence identity over ≥98% total length with previously identified variants. This results in a continually expanding catalogue of the diversity of all loci found in *Neisseria*. Gene-based comparisons can then be undertaken using the Genome Comparator tool within BIGSdb, which compares isolates using any number of loci predefined in the database or a reference genome. Through the use of this gene-by-gene approach to genome analysis, any combination of genes of interest can be compared between isolates ranging from the comparison of single loci to analyses of whole genomes resulting in a genome-wide MLST profile i.e. wgMLST (Maiden *et al.*^2013^). The benefit of this gene-by-gene approach being that the functional significance of genetic diversity can be rapidly deduced and compared between datasets.

### WGS analysis tools

PubMLST is particularly useful for the gene-by-gene annotation, comparison and analysis of WGS data; however, additional tools are available that facilitate the analysis of larger datasets including several thousand isolates. Genome alignment and phylogenetic representation can become computationally intensive in the *Neisseria* genome where genomic rearrangements, duplications, deletions and insertions frequently occur resulting in a genome formed of conserved regions interspersed with highly diverse regions (Hao *et al.*^2011^). Tools such as Gubbins (Genealogies Unbiased By recomBinations In Nucleotide Sequences) can be used to identify, and exclude from phylogenetic analysis, loci subject to recombination effectively resulting in the analysis of the core non-recombinant genome which can be further analysed using tools such as ClonalFrameML (Croucher *et al.*^2015^). Alternative tools including single nucleotide polymorphism (SNP) typing methods are also available. Read-based mapping algorithms carry out assembly-free analyses using a finished reference genome and a sensitive read mapper such as BWA or Smalt followed by a variant caller such as samtools/bcftools and variant filter methods (Li and Durbin 2009; Li *et al.*^2009^). These methods require read data that are not always available and can be substantially larger than assembled genomes. In addition, read mapping can be sensitive to contaminants, misalign repetitive sequences and introduce bias in phylogenetic reconstructions. This form of analysis is also dependent on the selection and availability of an appropriate reference genome.

K-mer matching can be used to identify and/or cluster homologous genomic sequence data and has recently been extended to detect SNPs within large datasets. This has been achieved through the development of the software kSNP which identifies odd-length k-mers that match all but the central position (Gardner, Slezak and Hall 2015). The location of the putative SNPs can then be determined by mapping matched k-mers back to a reference genome. kSNP therefore avoids the use of whole-genome alignments and has the added benefit of accepting both assembled or short read data as input files.

Although pattern-based approaches such as that employed by kSNP have been found to closely reconstruct phylogenetic trees and are superior in this respect to other alignment-free methods, accurate phylogenetic inference can only be reliably obtained through the generation of maximum-likelihood distances computed from multiple sequence alignments (Hohl and Ragan 2007). Methods that combine whole-genome alignment with read-mapping may overcome problems associated with sensitivity and accuracy, whilst still allowing the rapid analysis of large datasets: the software Parsnp has been specifically designed for this purpose (Treangen *et al.*^2014^). Parsnp identifies maximal-unique matches among assembled genomes to both recruit similar genomes and anchor the multiple alignments (Treangen *et al.*^2014^). Core-genome alignments, variant calls and a SNP tree are then generated which can be visually explored using the accompanying software Gingr. For example, Parsnp was recently used to analyse WGS data from the bacterial pathogen, *Streptococcus agalactiae.* In this study, maximum-likelihood trees were generated based on concatenated SNPs from aligned core genomes revealing the occurrence of recombination events between ST23 and ST17 *S. agalactiae* isolates and the emergence of a serotype IV ST-425 lineage (Campisi *et al.*^2016^).

The principle underlying all of these methods is that the core genome, which includes genes present in all isolates from one species, will predominantly contain essential genes vertically inherited from the common ancestor and with a high signal-to-noise ratio. These will thus allow accurate representations of WGS data while avoiding difficulties with aligning and interpreting the evolution of complex and diverse genomic regions. Using a combination of methods and tools including the BIGSdb Genome Comparator and Roary (Page *et al.*^2015^), core genomes have been characterised and defined in PubMLST at the *Neisseria* genus level (*Neisseria* genus cgMLST) (Bennett *et al.*^2010^), the species level for the meningococcus (*N. meningitidis* cgMLST) (Bratcher *et al.*^2014^) and the gonococcus (*N. gonorrhoeae* cgMLST) (Harrison *et al.*^2017^) as well as the lineage-specific level (Harrison *et al.*^2015^). The genus-wide availability of loci in PubMLST combined with multiple core genome catalogues will ultimately allow genomic differences between species to be identified. For example, 1605 core loci have been characterised in the meningococcus with a total of 1653 core loci defined in gonococci. Comparison of loci from these core datasets has revealed that 1395 of these were in common to both species leaving 210 loci unique to meningococci and 258 loci distinct to gonococci (Harrison, O.B. unpublished). Further analyses of these loci may unravel some of the fundamental differences observed in both species including the distinct pathologies caused.

## APPLICATIONS AND IMPLICATIONS OF NGS DATA FOR *NEISSERIA*

### Effective and robust diagnostics using WGS data

Major benefits of WGS data in the *Neisseria* community have included *N. meningitidis* outbreak detection (Mulhall *et al.*^2016^), *N. meningitidis* and *N. gonorrhoeae* isolate characterisation and disease surveillance (Lucidarme *et al.*^2015^; Jacobsson *et al.*^2016^; Stefanelli *et al.*^2016^), and *Neisseria* taxonomy using ribosomal MLST (Bennett *et al.*^2012^, ^2014^; Bennett, Jolley and Maiden 2013). A major achievement in particular has been the publicly available online database containing WGS from invasive meningococcal isolates originating from England and Wales and dating from 2010 to the present providing data to the *Neisseria* community in almost real-time (MRF-MGL). In addition, there is the possibility in the near future for antimicrobial resistance profiles to be detected in WGS data from *N. gonorrhoeae* (Harrison *et al.*^2016^; Abrams and Trees 2017; Eyre *et al.*^2017^). WGS also enables species identification through quantitative assessment of genome-wide similarity between a new isolate and a curated multispecies reference collection, using statistics such as average nucleotide identity (Richter and Rossello-Mora 2009). Faster algorithms such as MASH allow this approach to be extended to much larger reference collections (Ondov *et al.*^2016^). In addition, WGS data have recently been used to detect novel genetic mutations which may have led to the dissemination of new invasive meningococci or antimicrobial-resistant gonococci (Grad *et al.*^2014^; Taha *et al.*^2016^; Toh *et al.*^2017^). Using WGS data, there is also the potential to combine pathogen and patient genomic sequence data to better understand patient response and susceptibility to infection (Davila *et al.*^2010^; Renner *et al.*^2012^).

A benefit of WGS can be through the provision of comprehensive information on bacterial populations, including the analysis of complex genomic regions that previously required several independent PCR- and sequencing-based assays (Dolan Thomas *et al.*^2011^; Wang *et al.*^2011^, ^2012^). This in turn can, for example, accelerate the time required for species identification and improve clinical diagnosis including the provision of subtyping information (Jolley *et al.*^2012^), particularly in clinical specimens where viable cultures could not be obtained (Joseph *et al.*^2016^). Indeed, the use of molecular-based approaches to identify both meningococci and gonococci has been increasingly employed with detection of gonorrhoea in the UK now routinely performed using nucleic acid amplification tests, while over half of the invasive meningococcal disease (IMD) cases in the UK between 2009 and 2010 were diagnosed solely by PCR (Heinsbroek *et al.*^2013^; Mohammed *et al.*^2015^). A substantial benefit of whole genome sequencing will therefore be in the ability to improve diagnosis in large numbers of isolates with low cost per sample while also providing genomic sequence data for additional analyses (Jolley and Maiden 2013; Maiden and Harrison 2016), all of which prior to WGS would have been cost-prohibitive.

An additional application of WGS in clinical diagnostics is through the enhanced surveillance of *N. meningitidis* capsular groups associated with IMD. Conjugate polysaccharide vaccines have successfully reduced the burden of meningitis and septicaemia globally. Such vaccines can, however, lead to the emergence of capsule replacement, as seen with *Streptococcus pneumoniae* (Wyres *et al.*^2013^). In addition, through immune selection, meningococci with capsule types included in a vaccine formulation may acquire capsule genes from non-vaccine isolates giving rise to new variants through capsule switching. The continued surveillance of serogroups associated with IMD is therefore essential to monitor such events which may not be apparent using conventional seroagglutination and PCR methods (Mustapha *et al.*^2016^). Serogroup B meningococcal disease is the only major disease-associated capsular group of meningococci for which a polysaccharide conjugate vaccine is unavailable; therefore, multicomponent protein-based vaccines that include outer membrane proteins have been developed (Giuliani *et al.*^2006^; Bambini *et al.*^2009^) with WGS data facilitating the assessment of antigen diversity among broad *N. meningitidis* isolate collections (Brehony *et al.*^2015^). Two such vaccines, Bexsero^®^ and Trumenba^®^ have recently been licensed for use in the UK and the USA, and post-implementation surveillance of IMD meningococci, including characterisation of vaccine antigens, is essential to assess the effectiveness of such vaccines. A Bexsero Antigen Sequence Type (BAST) scheme, based on deduced peptide sequence variants from the antigen targets included in Bexsero (fHbp, PorA, NHBA and NadA) has been implemented in the PubMLST database allowing vaccine antigen variants to be characterised (Brehony *et al.*^2016^). Results from this study, which included invasive meningococci isolated in Great Britain and Ireland, showed that BASTs were strongly associated with serogroup and clonal complex and demonstrated a significant increase in BAST-2 associated with the increased prevalence of serogroup W clonal complex 11 meningococci.

### Recommendations and use of nomenclature

The effective use of WGS data for public health purposes requires the continued development of standardised software tools and effective data sharing networks to ensure that clear and robust results can be reported and compared globally. The practice of grouping and naming organisms on the basis of genotypic and phenotypic similarity facilitates communication and decision making. A principle of biological systematics is that biological names reflect evolutionary lineages and are therefore applied to clades (i.e. monophyletic groups). Due to the clonal reproduction of bacteria, clades can be identified even within species, although genetic recombination mediated by horizontal gene transfer means that clonal relationships do not necessarily describe the origins of the bacterial genomes over longer periods of time (Spratt 2004).

During outbreaks, pathogenic isolates tend to be grouped together on the assumption of sharing a recent common ancestor, representing one or more virulent strains circulating in the affected community. The complexity of genetic changes present, however, particularly when genetic differences occur in genes present only in some isolates (the accessory genome), demonstrates the challenges of interpreting genetic variation among isolates recovered from a single outbreak (Bergholz *et al.*^2015^). Genomic changes can occur even during a single case of IMD in one patient although evidence for concomitant within-host evolution of invasive phenotypes has not been detected so far (Omer *et al.*^2011^; Klughammer *et al.*^2017^; Lees *et al.*^2017^). This therefore introduces particular issues such as what should be the lowest cut-off value in genomic changes for transmission to be accurately detected (Hatherell *et al.*^2016^)? In addition, WGS analyses should always consider the possibility that patients may be co-infected with two or more distinct genotypes, particularly in the case of *N. gonorrhoeae* (Martin and Ison 2003). Such narrowly defined outbreak isolates will, however, only be relevant for a short time, as they will either become extinct or undergo further diversification.

Phylogeny-based definitions can account for the diversification of an outbreak strain over time, for example, the ‘Hajj clone’ from the Saudi Arabian meningococcal outbreak of 2000 (Lucidarme *et al.*^2015^; Mustapha *et al.*^2015^; Retchless *et al.*^2016^), but most isolates will not belong to a clade with a definable origin. A generalisable and stable nomenclature that facilitates communication may be best achieved by using an operational definition based on simple genomic similarity measures, which can serve as a surrogate for phylogenetic relationships among closely related strains (CDC unpublished data). A widely useful nomenclature scheme will need to include multiple ranks of within-species diversity to allow the rapid communication of genomic similarity for a variety of purposes, including short-term outbreak investigation, long-term surveillance programs and evolutionary studies. The PubMLST database currently performs genome clustering using a single-linkage algorithm based on cgMLST profiles. This precomputes groups of similar genomes based on allelic mismatch thresholds of 200, 100, 50 or 25 loci to at least one other member of the group. This clustering does not produce a stable nomenclature, due to the transient nature of groups which can merge as data are added, but will readily facilitate the identification of similar isolates in outbreaks or for wider epidemiological purposes. For a stable nomenclature, especially required for long-term surveillance, it would be possible to cluster based on thresholds of allelic differences to defined central cgMLST genotypes in the same way that standard MLST-based clonal complexes are defined for *Neisseria*.

## FUTURE PROSPECTS

### Challenges and prospects of functional genomic data integration

The collection of approaches referred to as ‘omics’ spans an increasing variety of fields such as genomics (the quantitative study of protein coding genes, regulatory elements and non-coding sequences), transcriptomics (RNA and gene expression), proteomics (protein abundance) and epigenomics (DNA methylation patterns). Ongoing advances in high-throughput sequencing methods facilitate such analyses, and will further our understanding of *Neisseria* infection and biology. For example, distinct base modifications implicated in epigenetic modifications can be determined in assembled WGS data using single molecule, real-time (SMRT) genome sequencing technology. Using SMRT, the recognition sites for three key *N. meningitidis* N(6)-adenine DNA methyltransferases (ModA11, ModA12 and ModD1) were identified unravelling global methylation patterns which can result in phase-variable changes in meningococcal gene expression (Seib *et al.*^2015^).

Conventional deep transcriptome sequencing (RNA-seq) as well as transcriptome analyses using tiling arrays have already enriched our understanding of meningococcal and gonococcal transcriptome dynamics and organisation. In particular, these technologies have allowed the genome-wide identification of small ncRNAs in meningococci and gonococci demonstrating their important contribution in *Neisseria* physiology (Mellin *et al.*^2010^; Huis in 't Veld *et al.*^2011^; Wachter and Hill 2015), including the regulation of large metabolic adaptations (Pannekoek *et al.*^2017^), survival in human blood (Del Tordello *et al.*^2012^) as well as during infection *in vivo* (Fagnocchi *et al.*^2015^; McClure *et al.*^2015^) and enhancement of pilin variation in both meningococci and gonococci (Cahoon and Seifert 2013; Tan *et al.*^2015^). The still expanding array of high-throughput sequencing-based technologies (reviewed in Barquist and Vogel 2015) is adding novel data on *Neisseria* genome architecture and gene expression regulation at an unprecedented detail and with increasing pace. These technologies include differential RNA-seq (dRNA-seq) (Sharma *et al.*^2010^) and Term-seq (Dar *et al.*^2016^) which allow the genome-wide identification down to the single nucleotide level of transcript boundaries and operons as well as the reliable identification of novel transcripts including trans-acting ncRNAs and cis-regulatory RNA elements such as riboswitches or attenuators. Accordingly, using dRNA-seq, Heidrich *et al.* (2017) mapped 1625 transcriptional start sites in the genome from *N. meningitidis* strain 8013 along with 64 ncRNAs and revealed an unexpected paucity of classical σ70-type promoters. Furthermore, Zhang *et al.* (2013) demonstrated that meningococci possessed the most streamlined CRISPR/Cas system characterised to date, thus limiting natural transformation—the primary source of genetic variation in this species.

The specific interactions between transcripts and nucleic acid-binding proteins such as Hfq can be studied on a genome-wide basis under various experimental conditions with new sequencing-based technologies such as RIP-seq (Sittka *et al.*^2008^), CLIP-seq (Holmqvist *et al.*^2016^) or Grad-seq (Smirnov *et al.*^2016^), which allow transcriptome-wide mapping of *Neisseria* RNA-binding protein target sites to rapidly describe functional RNA landscapes. High-throughput sequencing has been further used to modify classical genome-wide transposon mutagenesis approaches in order to determine gene function in various experimental conditions. For example, Capel *et al.* (2016) were the first to use transposon insertion site sequencing (Tn-seq) to comprehensively identify 228 protein-coding genes as well as 33 ncRNA candidates required for meningococcal colonisation of human cells. A recently developed sequencing technology is dual RNA-seq (Aprianto *et al.*^2016^; Westermann *et al.*^2016^) which allows the simultaneous profiling of all RNA classes in both bacteria and the eukaryotic host cell, and thus promises to deliver novel insights into *Neisseria* infection biology. Given the high genetic diversity of the *Neisseria*, however, it remains to be shown whether the results obtained for one particular genotype will be representative of the entire species. In fact, it is known that the high genetic diversity observed might not be entirely neutral (Buckee *et al.*^2008^) and may result in phenotypic differences at the transcriptomic (Joseph *et al.*^2010^) and likely metabolic level therefore affecting virulence in a strain-dependent manner (Schoen *et al.*^2014^). In support of this hypothesis, a recent systems biology analysis, comparing the transcriptomes from a carried and invasive-associated meningococcus in conditions mimicking infection, indicated that inactivating mutations in amino acid metabolism genes were buffered at the transcriptional level. Consequently, while both meningococci were able to grow in human blood, they showed significant differences in the expression of numerous virulence-associated determinants suggesting that meningococcal virulence was linked to transcriptional buffering of cryptic genetic variation in metabolic genes (Ampattu *et al.*^2017^).

**Figure 4.**
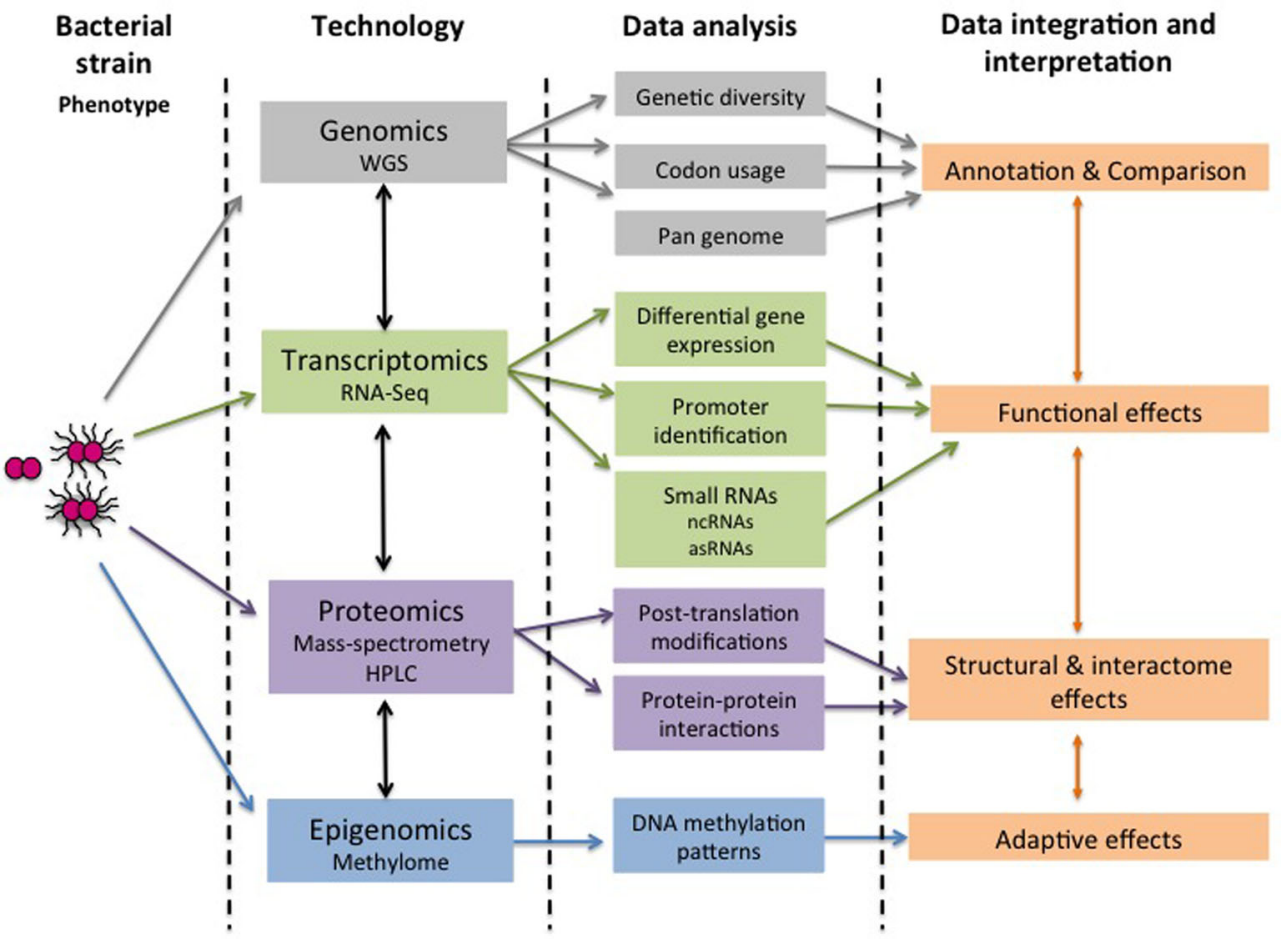
Omics platforms. Flow diagram depicting available Omics approaches some of which have been used in *Neisseria* research. Possible ways in which these approaches can be associated are also indicated.

All these genome-wide analyses may generate more data than is analysed and published; however, these data are not lost but can be used to improve functional genome annotation and provide the raw material for integrative analyses that in turn can guide functional and molecular studies. The feasibility and power of combining genomic data with computational simulation to predict phenotype from genotype has already been demonstrated for a number of model species such as *Mycoplasma genitalium* (Karr *et al.*^2012^) or *Escherichia coli* (Carrera *et al.*^2014^). Dedicated databases and software exist for the storage and analysis of *Neisseria* WGS from a large number of strains such as BIGSdb (Jolley and Maiden 2010) or NeMeSys (Rusniok *et al.*^2009^). Future planned developments of BIGSdb will allow the arbitrary storage and querying of any number of sparsely-populated fields facilitating the integration of complete phenotypic and transcriptomic data with existing genome and provenance information. In addition, a computational genome-scale metabolic model has already been established and experimentally verified for *N. meningitidis* MC58 (Baart *et al.*^2007^; Mendum *et al.*^2011^). Given that such genome-scale models are able to highlight strain-specific adaptations to different nutritional environments corresponding to bacterial pathotypes (Monk *et al.*^2013^), it is anticipated that computational model-based analyses of the huge omics datasets becoming available in the near future will be key in understanding the impact of genetic variation in both meningococcal and gonococcal phenotypes. Integration of the large and heterogeneous omics datasets and making these accessible for comprehensive computational analyses will therefore be instrumental in enriching our understanding of *Neisseria* biology (Fig. 4).

## CONCLUSIONS

From the elucidation of the structure of DNA to the publication of the first bacterial genome, our fascination with DNA and the genome it encodes shows no sign of diminishing. The impact genetic differences may have in a bacterial population ranging from the ability to successfully avoid the immune response to becoming antimicrobial resistant has become more carefully considered and understood. It is only through the comparison and analysis of large-scale bacterial populations, however, that we will be able to fully appreciate the consequences of these. Future challenges now lie in establishing ways in which the genome can be linked with the transcriptome, proteome and epigenome, ultimately resulting in a connection between the genotype and the phenotype and unravelling the complexity of bacterial infections.
